# Bioactivity and toxicity of coumarins from African medicinal plants

**DOI:** 10.3389/fphar.2023.1231006

**Published:** 2024-01-10

**Authors:** Godwin Anywar, Emmanuel Muhumuza

**Affiliations:** Department of Plant Sciences, Microbiology and Biotechnology, College of Natural Sciences, Makerere University, Kampala, Uganda

**Keywords:** coumarins, bioavailability, safety, therapeutic, medicinal plants, drug discovery

## Abstract

**Introduction:** Coumarins are naturally occuring metabolites from plants and a few micro-organisms. They have been widely used in the food and drug industry in their natural or synthetic forms. Numerous coumarins possess several biological activities such as anti-inflammatory, anti-ulcers, anti-tumour, anti-microbial, anti-coagulant. The aim of this study was to assess the bioactivity, and toxicity of coumarins from African medicinal plants.

**Methods:** We searched online databases and search engines such as PubMed, Google Scholar and Web of Science for key terms such as coumarins, toxicity, bioavailability, bioactivity with appropriate Boolean operators. Only full-length research articles published in English between 1956 to 2023 were reviewed.

**Results:** We recorded 22 coumarins from 15 plant species from Africa. Most of the plant species (33%) were from North Africa. These were followed by East Africa at 21%, then West, and Central Africa at 18.2% each. Most of the coumarins (21.3%) were isolated from the entire plant and the leaves (19.1%) and most of them (46.7%) had some antimicrobial activity. Five coumarins viz osthole, pseudocordatolide C & calanolide, chartreusin and esculetin had either antitumor or anticancer activity. Six coumarins had varying levels and types of toxicity ranging from inhibiting blood clotting as anticoagulants, to cytotoxic effects, causing hyperventilation, tremor, & photophobia, pulmonary haemorrhage, carcinogenic activity, severe neurotoxicity, hepato- and phototoxicity.

**Conclusion:** Several African medicinal plants are sources of various coumarins that possess several biological activities as well as toxicities. This calls for more research into their safety and efficacy because of their wide spread applications as therapeutic agents.

## 1 Introduction

Coumarins are naturally occurring metabolites in a variety of plants, micro-organisms and in some animal species (Perone, 2016). Coumarin (1,2-benzopyrone; 2H-1-benzopyran-2-one; cis-o-coumarinic acid lactone) is also known as benzopyrone or coumarinic anhydride and tonka bean camphor. It is a white crystalline solid, belonging to the class of lactones. It consists of an aromatic ring fused to a condensed lactone ring (Venugopala et al., 2013).

**FIGURE 1:**
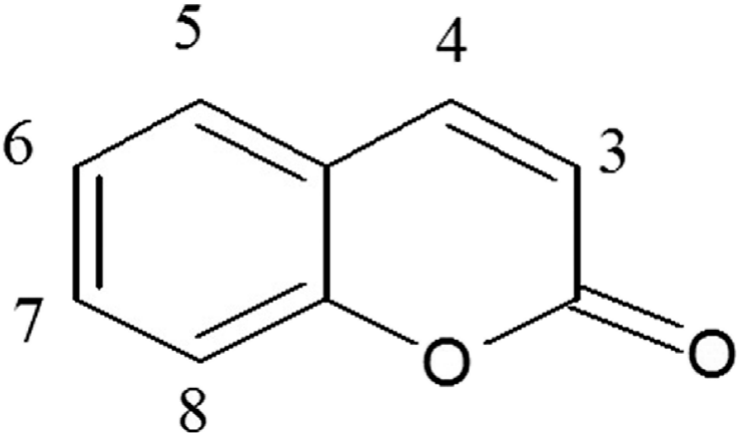
Structure of simple coumarins.

Coumarins usually occur in plants as glycosides and esters but mostly occur in free form. Coumaric metabolites are lactones of 2-coumaric acid (2-hydroxy-Z-cinnamic acid) and are constructed by a benzene ring fused to an α-pyrone ring (Figure 1) (Matos et al., 2015). Coumarins are very complex and diverse metabolites. Naturally occurring coumarins may be categorized as coumarins, isocoumarins, furanocoumarins, pyranocoumarins, biscoumarins, and phenylcoumarins (Lacy and O’kennedy, 2004; Zhu and Jiang, 2018) and synthetic pyrone-substituted coumarins (Lacy and O’kennedy, 2004).

The chemical formula of coumarins is C_9_H_6_O_2_, and they can exist in various isomeric forms due to differences in the substitution pattern on the benzene and the pyrone ring (Wu et al., 2009). The presence or absence of particular functional groups confers the coumarin its name and properties, such as simple coumarins which are the basic coumarin metabolites with no additional substituents. These include hydroxycoumarins like scopoletin and umbelliferone, methoxycoumarins like esculetin and 7-methoxycoumarin and alkyl coumarins like daphnetin and aesculetin (Wu et al., 2009). Coumarins are variably soluble in most organic solvents or their combinations (Huang et al., 2015) but are freely soluble in ethanol, chloroform, diethyl ether and oils (Jung and Oh, 2011).

Coumarin occurs naturally in many plants and confers a pleasant spice aroma akin to vanilla (Egan et al., 1990). Coumarins were first isolated from *Dipteryx odorata* (Aubl.) Forsyth f (tonka beans) by Vogel in 1820 (Doctor et al., 2020). They also occur in high concentrations in some essential oils, such as *Cinnamomum aromaticum* (cinnamon bark oil) and *Cassia fistula* L. (cassia leaf oil). Coumarins have been identified in various plant species from many different families including the fruits of V*accinium myrtillus* L. (bilberry), *Rubus chamaemorus* L (cloudberry)*, Camellia sinensis* (L.) Kuntze (green tea), *Melilotus albus* Medik. and *Melilotus officinalis* (L.) Lam (sweet clover), *Galium odoratum* (L.) Scop(sweet woodruff), *Vanilla planifolia* Andrews (vanilla leaves and beans), *Lavendula officinalis* subsp. angustifolium (lavender) and Myroxylon *pereirae* (L.) Harms (balsam of Peru) (Kostova et al., 2011; Kicel and Wolbis, 2012). A broad spectrum of coumarins present in both free state and as glucosides have also been found in many other plant species. Several coumarins have been identified, principally as secondary metabolites in green plants, but the exact number is hard to determine due to synthetic coumarins created regularly (Kostova, 2005).

Most commercially used coumarins were synthesized from salicylaldehyde (DeGarmo and Raizman, 1967). High-grade coumarin is still isolated from tonka beans using various methods such as hot water steeping and sub critical water extraction (Doctor et al., 2020). Coumarins are extremely variable in structure leading to various pharmacological activities (Lacy and O’kennedy, 2004). The simplicity and versatility of the coumarin scaffold makes it a convenient starting-point for a wide range of applications (Stefanachi et al., 2018; Annunziata et al., 2020).

### 1.1 Industrial applications and uses of Coumarins

Although coumarins are widely distributed in the plant kingdom, most of the commercially used ones have been synthetically produced for many years. Coumarins have several applications such as in the perfumery, cosmetic and related industries (Givel, 2003). Large quantities of coumarin, mostly associated with vanillin, are used in the food industry for flavouring chocolates, baked goods, and in flavoured beverages (Givel, 2003). However, since 1954, synthetic vanillin use as a direct additive in tobacco products has been suspended in the United States by the FDA due to the production of hazardous compounds when burnt (Riveiro et al., 2010; Sarker and Nahar, 2017; Prusty and Kumar, 2020). Because of their unique sweet note and stability, coumarins have long been recognized as an important raw material in the fragrance industry as odour-enhancers. They help to achieve a long-lasting effect when combined with natural essential oils (Doctor et al., 2020). As such, they are widely used in hand soaps, detergents, lotions and perfumes, in tobacco to enhance its natural aroma, in toothpastes, antiperspirant deodorants, creams, hair sprays, shampoos and many other household products (Perone, 2016). Large quantities of coumarins are used in rubber and plastic materials and in paints and sprays to neutralize unpleasant odours (Kirsch et al., 2016). Coumarins also have significant uses in electroplating, mostly in the automotive industry, to provide high polished quality chrome-plated steel. However, this use has been on the decline (Kirsch et al., 2016).

### 1.2 Medicinal applications of coumarins

Coumarins have several pharmacological properties with applications in the medical field (Skalicka-Woźniak et al., 2016), as a cardiovascular targeting drug (Najmanova et al., 2015), and as a cancer screening molecule and therapy (Lacy and O'Kennedy, 2004). Coumarins possess both immunomodulatory and antitumor activity (Riveiro et al., 2010). They have been recommended for treatment of a number of clinical conditions, including high protein oedema and brucellosis (Luszczki et al., 2009; Riveiro et al., 2010; Raja et al., 2011). Coumarins such as osthole have undergone clinical trials for treatment of lymphoedema (Farinola and Piller, 2005), following breast cancer treatment and in treatment of lung and kidney cancer and melanoma alone or in combination with cimetidine (Riveiro et al., 2010; Prusty and Kumar, 2020). Coumarins have also been used for prevention of dental caries (Perone, 2016). Coumarins have been tested for treatment of schizophrenia, microcirculation disorders and angiopathic ulcers (Skalicka-Woźniak et al., 2016). The vitamin K epoxide reductase (VKORC1), the key enzyme of the vitamin K cycle is the molecular target of coumarins (Oldenburg et al., 2007). Thus, the objective of this study was to assess the bioactivity, and toxicity of coumarins from African medicinal plants.

## 2 Methods

We performed a literature search using PubMed, Google Scholar and Web of Science. We searched for terms such as coumarins, toxicity, bioavialability, bioactivity with appropriate Boolean operators. Only full-length articles published in English between 1956–2023 were reviewed. The review was limited to African medicinal plant species only. We used only original research articles or review papers and excluded results from *in silico* studies.

## 3 Results

We recorded 22 coumarins that were isolated from 15 plant species (Table 1). Most of the plant species (33%) were from North Africa, followed by East Africa (21%), then West, and Central Africa at 18.2% each. Most of these coumarins (21.3%) were isolated from the whole plant. These were followed by the leaves (19.1%), then seeds, fruits and roots each at 12.8%. However, only two plant species *Mammea africana* Sabine and *Tetrapleura tetraptera* (Schumach. & Thonn.) Taub. had a more restricted native range to Africa. The rest of the plant species were more widely distributed in different parts of the world. The distribution of the plant species was checked with the Kew database at https://powo.science.kew.org. All the coumarins showed multiple bioactivities. For instance, esculin had more than six bioactivities. Ten different coumarins had antimicrobial activity including antibacterial, antiviral, antifungal or anti-parasitic activity. Bicoumarin/Biscoumarin and dicoumarol had anticoagulant activity while three coumarins: esculetin, scopoletin, dicoumarol had anti-inflammatory activity. Five coumarins: osthole, pseudocordatolide C and calanolide, chartreusin and esculetin had either antitumor or anticancer activity.

**TABLE 1:** Bioactivity of coumarins from African medicinal plants.

Coumarins	Plant source & (family)	Type of extraction and compounds recovery	Part used	Country/Area of collection	Bioactivity	Measure of activity
1. Bicoumarin/Biscoumarin	*Melilotus officinalis* (L.) Lam. (Fabaceae)	Ultrasonic bath and ultrasound-assisted extraction using a rotavap (Zhang et al., 2018).	L	South, North Africa. Widely distributed globally	Anticoagulant, antimicrobial	Vitamin K-blockers. Act as anticoagulants by inhibiting the cyclic inter-conversion of both vitamin K, vitamin K epoxide (Eason and Spurr, 1995; Hirsh et al., 2001; Sun et al., 2020).
2. Dicoumarol					Anti-inflammatory, Anticoagulant	Vitamin K-blockers that act as anticoagulants *via* inhibiting the cyclic inter-conversion of both vitamin K, vitamin K epoxide (Hirsh et al., 2001; Jung and Oh, 2011; Kubrak et al., 2017).
3. Psoralen	*Fatoua pilosa* Gaudich. (Moraceae) (Chiang et al., 2010).	Organic solvent extraction, column chromatography & Medium performance liquid chromatography (Hsia et al., 2019).	Wp	West, central, East Africa. Grows primarily in the wet tropical biome	Antifungal, Antimycobacterial against *Mycobacterium tuberculosis* H37Rv, Anti-HIV	MIC = 42 μg/mL against *M. tuberculosis* H37Rv (Jadhav et al., 2017). 50 μM taken with imperatorin inhibited HIV-1 replication in HeLa cells (Sancho et al., 2004).
4. Bergapten					Anti-tumor activity against breast cancer cells, antimycobacterial against *Mycobacterium tuberculosis* H37Rv, antimicrobial	Bergapten increased NF-Y nuclear translocation through p38 MAPK activation hence treatment induced a block in the G0/G1 phase and increased mRNA and protein levels of p53 and p21waf (Panno et al., 2009; Santoro et al., 2016).
5. Dihydromammea	*Mammea africana* Sabine (Guttiferae)	Methanol extracts separated by liquid–liquid extraction, & open column chromatography (Chakthong et al., 2012; Crichton and Waterman, 1978).	St/Sd	West Africa. Native range is W. Tropical Africa to SW Uganda and Angola	Antihypertensive, anti-hyperglycemic	200 mg/kg day showed anti-hypertensive activity when administered orally in rats together with 40 mg/kg day L-NAME (Nguelefack-Mbuyo et al., 2008).
6. Osthole	*Ferulago galbanifera* (Mill.) W.D.J.Koch (Apiaceae)	Organic solvent extraction and column chromatography (Karakaya et al., 2019).	R	Morocco, but more widely distributed in the Mediterranean	Antioxidant, antimicrobial, antifungal, antitumor, & anticonvulsant, multiple sclerosis	Doses of 253–639 mg/kg, bw in mice showed significant antioxidant activity (Liu et al., 2012; Yang et al., 2010). In convulsant studies, scores were significantly reduced in early treated EAE mice compared to untreated EAE mice (1.42 ± 0.20 vs3.57 ± 0.30) (Teng et al., 1994; Zhang et al., 2015).
7. Anthogenol	*Aegle marmelos* (L.) Corrêa (Rutaceae)	Organic solvent extraction & TLC (Chakthong et al., 2012).	Fr	Tropical Africa	Antibacterial, Anti-emetic	Effective against *Enterococcus* bacteria strains (Kubrak et al., 2017)
8. Pseudocordatolide C & calanolide	*Calophyllum lanigerum* Miq. (Calophyllaceae)	Organic extraction and purification using HPLC (McKee et al., 1996).	L	Central & West Africa	Anticancer, Anti-HIV, Antidermatitic	IC_50_ = 290–351 mol ratio/32 pmol TPA was effective against leukemia progression (Nahar et al., 2020), IC_50_ = 120 μg/mL against breast cancer in cell lines (Sancho et al., 2004).
9. Phellodenol A	*Phellodendron amurense* Rupr. (Rutaceae) (Wu et al., 2003).		L	East Africa	Antimycobacterial against *M. tuberculosis*	Effective dose at 60 mg/mL in mice (Reddy et al., 2021).
10. Chartreusin	*Dendrobium officinale* Kimura & Migo (Orchidaceae) (Zhao et al., 2020).		Fr & Sd	Tropical Africa	Cardioprotective, anti-tumor, antimicrobial	Cytotoxic against Hep3B2.1-7 (IC_50_ = 18.19 µM) & H1299 (IC_50_ = 19.74 µM) cancer cell lines (Nahar et al., 2020). Antibacterial against *S. aureus* (IC_50_ = 23.25 µM) in mice (Venugopala et al., 2013).
11. Fraxidin/Fraxin/Fraxacin	*Fraxinus chinensis* subsp. *rhynchophylla* (Hance) A.E.Murray (Oleaceae) (Qu et al., 2021).		B	Algeria, Morrocco	Antiadipogenic, antihyperglycemic	Dosages between 30 and 90 mg/kg in mice decreased TG, CH, LDL, IL-1, IL-6, ICAM-1, NO, levels (Hirsch et al., 2002; Dabur et al., 2018)
12. Calanolide A and B	*Calophyllum lanigerum* Miq. (Clusiaceae)		L	Central & West Africa	Anti-HIV	IC_50_ = 130 µM in suppressing HIV1 reverse transcriptase (Buckheit et al., 1999). Highly effective against AZT-resistant G-9106 strain of HIV-1 & pyridinone-resistant A17 strain (Boyer et al., 1993).
13. Visnadine/Visnagin	*Ammi daucoides* Gaertn. (Apiaceae)		Fl & Fr	North Africa	Muscle dilator	Pectoris, peripheral and coronary muscle vasodilator (Khalil et al., 2020).
14. Esculin	*Fatoua pilosa* Gaudich. (Moraceae)		Wp	Central, West & East Africa Algeria, Morocco	Antiadipogenic Antioxidant	Concentrations of 30–90 mg/kg in mice decreased TG, CH, LDL, IL-1, IL-6, ICAM-1, NO, levels. (Hirsch et al., 2002; Venugopala et al., 2013; Dabur et al., 2018).
	*Fraxinus chinensis* subsp. *rhynchophylla* (Hance) A.E.Murray (Oleaceae)					
15. Esculetin	*Bougainvillea spectabilis* Willd. (Nyctaginaceae)		Sd & St	East Africa but more widely distributed in Asia and South America	Neuroprotective, antioxidant, antiadipogenic, anticancer, anti-inflammatory	Intracerebroventricularly effective, 30 min after administration at a concentration of 20 μg/mL in mice (Wang et al., 2012).
	*Cichorium intybus* L. (Cichoriaceae)					
16. Scopoletin	*Fatoua pilosa* Gaudich. (Moraceae)	Organic solvent extraction and column chromatography (Marealle et al., 2023).	Wp F	Central, West & East Africa Restricted to tropical Africa	Antimycobacterial, hypotensive, muscle relaxant Anti-inflammatory	MIC readings of 58.3 (Reddy et al., 2021) and 42 μg/mL against *M. tuberculosis* H37Rv (Chiang et al., 2010).
	*Tetrapleura tetraptera* (Schumach. & Thonn.) Taub. (Fabaceae)					T. tetraptera extract (50–800 mg/kg p.o.) produced dose-related, significant reductions (*p* < 0.05–0.001) of the fresh egg albumin-induced acute inflammation of the rat hind paw oedema compared to Diclofenac (100 mg/kg p.o.) (Ojewole and Adewunmi, 2004).
17. Grandivittin	*Ferulago galbanifera* (Mill.) W.D.J.Koch (Apiaceae)	Organic solvent extraction and purified by vacuum liquid chromatography (Ahmadi et al., 2016).	R	East Africa	Antibacterial against *Helicobacter pylori*	MIC = 125 ug/mL against *H. pylori* (Riveiro et al., 2010).
18. Aegelinol		Organic solvent extraction and column chromatography (Basile et al., 2009).			Antibacterial against Gram-positive and negative bacteria	MIC = 16ug/mL against *S. aureus*, *S. typhi*, *E. cloaca* 32ug/mL against *E. earogenes* (Riveiro et al., 2010; Venugopala et al., 2013).

**FIGURE 2:**
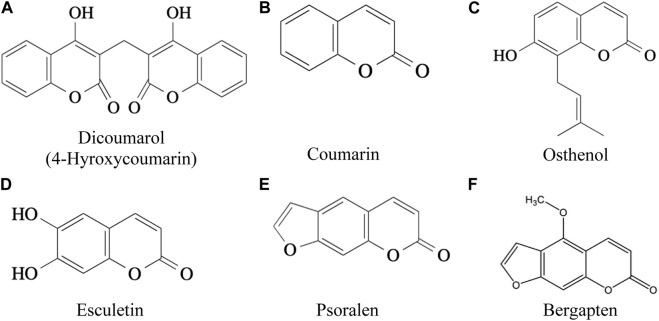
**(A)** Dicoumarol (4-Hyroxycoumarin) **(B)** Coumarin **(C)** Osthenol **(D)** Esculetin **(E)** Psoralen **(F)** Bergapten (Ulrich et al. 1999).

Six coumarins (dicoumarol, coumarin, osthole, esculetin, psoralen, bergapten, Figures 2A–F) had varying levels and types of toxicity ranging from excessive bleeding due to inhibition of blood clotting (anticoagulants), cytotoxic effects causing hyperventilation, tremor, & photophobia, pulmonary haemorrhage, carcinogenic activity, severe neurotoxicity, hepatotoxicity and phototoxicity (Table 2). Osthole, a natural coumarin isolated from dried fruits of *Cnidium monnieri* (L.) Cusson, had anti-allergic activity.

**TABLE 2:** Toxicity of coumarins derived from African plants.

Coumarin name	Plant source & (family)	Part (s)	Country	Toxicity	Measure/comment
1. Dicoumarol	*Melilotus officinalis* (L.) Lam. (Fabaceae)	L	South Africa	Anticoagulant Inhibits blood clotting leading to severe blood loss	Inhibits hepatic synthesis of coagulation factors, acting as a vitamin K antagonist (Yarnell and Abascal, 2009).
2. Coumarin	*Aegle marmelos* (L.) Corrêa (Rutaceae)	Fr	Nigeria	Cytotoxic effects	LD_50_ = 196–780 mg/kg bw with signs of liver toxicity (Mustafa et al., 2021)
3. Osthole	*Ferulago galbanifera* (Mill.) W.D.J.Koch (Apiaceae)	R	Morocco	Hyperventilation, acute neurotoxicity, tremor and photophobia	LD_50_ = 710 mg/kg bw in mice, 5–50 mg/kg in rats ED_50_ = 253–639 mg/kg and acute neurotoxicity at doses of 531–648 mg/kg (Shokoohinia et al., 2017), 5–50 mg/kg in rats (Liu et al., 2012).
	*Cnidium monnieri* (L.) Cusson (Apiaceae)				
4. Esculetin	*Bougainvillea spectabilis* Willd. (Nyctaginaceae)	Sd & St	East & West Africa	Carcinogen, mildly cytotoxic	A 20 mg/kg bw injection was lethal to rats after 24 h (Hazleton et al., 1956). Oral LD_50_ < 2000 mg/kg bw, intraperitoneal LD_50_ = 1,450 mg/kg bw (Eason and Spurr, 1995).
	*Cichorium intybus* L. (Nyctaginaceae)				
5. Imperatorin	*Angelica archangelica* L	Wp	North & South Africa	Severe neurotoxicity	TD_50_ = 329–443 mg/kg (Hazleton et al., 1956).
	*Angelica dahurica* (Hoffm.) Benth. & Hook.f. Ex Franch. & Sav. (Apiaceae)				
6. Psoralen	*Fatoua pilosa* Gaudich. (Apiaceae)	Wp	Central, West & East Africa	Hepatotoxicity	LD_50_ = 80 mg/kg bw in rats & 320 mg/kg in mice resulted into cholestatic liver injuries (Wang et al., 2013).
7. Bergapten	*Fatoua pilosa* Gaudich. (Apiaceae)	Wp	Central, West & East Africa	Phototoxic	Sun sensitivity to eyes and skin (Wagstaff, 1991).

Wp = Whole plant, L = leaves, Fr = Fruit, R = root, Sd = Seeds, St = Stem, bw = body weight.

## 4 Discussion

### 4.1 Antioxidant properties of coumarins

Osthole, esculin, dihydroxycoumarin and many other coumarins have shown beneficial biochemical profiles in relation to pathophysiological processes dependent upon reactive oxygen species due to the possession of amine, and hydroxy groups (Kostova et al., 2011). A study by Abdizadeh et al. (2020) evaluated the photoprotective activity of some hydroxycoumarins as compared with p-aminobenzoic acid (PABA) as a model sun screen. The activity could be related to their antioxidant actions which could minimize skin photoaging (Abdizadeh et al., 2020). Another study by Mitra et al. (2013) compared the antioxidative activities of seven hydrocoumarins with those of α-tocopherol for the oxidation of tetralin and linoleic acid in a homogeneous solution. Hydrocoumarins exhibited a higher induction period than α-tocopherol in both systems. However, the rate of oxygen absorption during the induction period for the latter was slower than that of the hydrocoumarins in both systems. In addition, 6,7-dihydroxy-4,4-dimethylhydrocoumarin showed less cytotoxicity toward human fibroblasts than 2, 6-di-t-butyl4-methylphenol (Kostova et al., 2011).

### 4.2 Antimicrobial activity of coumarins

Although different studies have confirmed the antimicrobial activities of various coumarins from plants (Vaou et al., 2021; Ojala et al., 1999; Karakaya et al., 2019), such studies are lacking of African medicinal plants. Some coumarin antibiotics of natural origin, although not necessarily from African plant species have been developed into modern antibiotics. These include NOVOBIOCIN™ and CLOROBIOCIN™ which are inhibitors of DNA gyrase. They both have a broad spectrum towards Gram-positive bacteria, including methicilin resistant strains of *staphylococci* species (Raja et al., 2011; Matos et al., 2012). This shows the potential of African plant species as a source of coumarin based drugs. Due to some limitations of these metabolites particularly with regard to solubility, toxicity and development of resistance, a novel series of coumarin analogues has been synthesized (Kirsch et al., 2016). The ester or carboxylic acid on the coumarin ring was found to be important for the inhibitory activity against Gram-positive and Gram-negative bacteria (Chiang et al., 2010; Annunziata et al., 2020). The presence of phenolic hydroxyl groups and or carboxylic acid was found necessary for enhanced activity against *Helicobacter pylori* (Venugopala et al., 2013).

### 4.3 Anti-inflammatory action of coumarins

Several coumarins like esculetin, dicoumarol isolated from plants or of synthetic origin possess significant anti-inflammatory and or analgesic activities (Kirsch et al., 2016). In a Quantitative Structure-Activity Relationship (QSAR) study for lead optimization in the design of coumarins as potent non-steroidal anti-inflammatory agents, the coumarin positions C4- and C7- contributed to the high activity (Poumale et al., 2013). Natural products such as esculetin, fraxetin, inhibit lipoxygenase and cyclooxygenase enzymic systems and neutrophil-dependent superoxide anion generation (Kontogiorgis and Hadjipavlou-Litina, 2005).

### 4.4 Antiviral properties of coumarins

This activity focuses essentially on the inhibition of HIV-1 protease (HIV-PR) and HIV-1 integrase (Sancho et al., 2004). PHENPROCOUMON™, warfarin and substituted 4-hydroxy-2-pyrone derivatives are actually referred to as the first generation of HIV-PR inhibitors (Hassan et al., 2016). Certain coumarin dimers, particularly those containing hydrophobic moieties on the linker, display potent inhibitory activity against HIV-1 integrase (Sancho et al., 2004). Calanolide A and B are semi-synthetic coumarins on market used as combination therapies for HIV. Psoralen was found to be a very potent anti-HIV drug during research but was discontinued for its high hepatotoxicity in mice (Sancho et al., 2004; Guo et al., 2020). Phenprocoumon emerged as a novel lead template possessing weak HIV protease inhibitory activity (Hassan et al., 2016). TRIOXSALEN™ was then developed and marketed as chemical derivative of psoralen despite its photosensitization of the skin (Wishart et al., 2018).

### 4.5 Antitumor/anticancer properties of coumarins

Osthole, umbelliferon, esculetin and geiparvarin all showed anti-tumor activity. Hydroxycoumarin might be a useful adjuvant for melanoma therapy because of its selective anti-proliferative agents that mediate apoptosis in renal carcinoma cells, through modulation of mitogen-activated protein kinases attributed to a nitro group in the aromatic ring (Kostova, 2005; Jung and Oh, 2011). In an attempt by Zhang et al. (2021) to develop novel antitumor candidates, a series of coumarin sulfonamides and amides derivatives were designed and synthetized. The majority of these metabolites showed good cytotoxic activity against MDA-MB-231 and kB cell lines (Zhang et al., 2021). Menezes and Diederich, (2019) conducted an extensive review on the role of natural coumarins as anticancer agents. They showed how coumarins modulate several targets such as carbonic anhydrase, Hsp90, histone deacetylase and topoisomerase enzyme in cancer cells leading to apoptosis.

### 4.6 Coumarins as enzyme inhibitors

Some natural and synthetic coumarins are cholinesterase inhibitors, which are a promising approach for the treatment of Alzheimer´s disease and possible therapeutic applications in the treatment of Parkinsons´s disease (Anand et al., 2012). A series of umbelliferon metabolites and synthetic coumarins were evaluated as inhibitors of monoaminooxigenase (MAO) and steroid 5α reductase type I in cell culture systems (Anand et al., 2012). Ether derivatives demonstrate MAO-B selectivity, and sulfonic ester derivatives demonstrate MAO-A selectivity by QSAR studies (Koyiparambath et al., 2021). Calanolide-A drugs are marketed for their ability to block activity of cytochrome P450 3A4, while ethyl biscoumacetate is a glutamine synthase inhibitor (Wishart et al., 2018; Sai et al., 1999).

### 4.7 Effect of coumarins on cardiovascular system

Many coumarins have different effects on blood. Most notable is warfarin, which has anti-coagulant properties (Hosseini et al., 2015). Warfarin drugs are used for the management of thromboembolism and pulmonary embolism with atrial fibrillation (Wishart et al., 2018). Ethyl biscoumacetate is a semi-synthetic compound used as anti-coagulant alternative to warfarin due to fewer side effects (Sun et al., 2020). Esculin and its metabolites are used as vasoprotective agents (Kostova, 2005). Moreover, other coumarins like biscoumarin/bicoumarin have similar activity to ethyl biscoumacetate and esculin (Sun et al., 2020; Najmanova et al., 2015).

### 4.8 Toxicity of coumarins

There is a large body of data on the toxicity of coumarin in experimental laboratory animals. Coumarins have hepatotoxic and carcinogenic properties *in vivo* in rodents and other mammalian species. These included adenomas and carcinomas of the liver, lungs and bile ducts and adenomas of the kidney and liver in rats and mice respectively. Carcinomas were found only at doses higher than 100 mg/kg body weight per day (Lake, 1999; EFSA, 2004). In humans, coumarins were approved for clinical use since the 1970s for treating various venous and lymphatic oedemas, as well as tumours including renal cell carcinoma (Marshall et al., 1994). However, their use was eventually discontinued and they were recalled from the market following the development of severe hepatotoxicity in patients after treatment (Cox et al., 1989; WHO, 1995). Coumarins are rapidly absorbed from the gastrointestinal tract after oral administration and extensively metabolized by the liver in the first pass, with only 2%–6% reaching the systemic circulation intact (Guo et al., 2020). Coumarins such as furanocoumarins are toxic to humans. Methoxypsoralen derivatives are potent photosensitizers. They can be activated by near-UV light becoming mutagenic, phototoxic and carcinogenic (Ojala et al., 1999; Ceska et al., 1997). Osthole had an acute intraperitoneal LD_50_ in mice of 710 mg/kg bw manifesting toxicity as hyperventilation, tremors, and photophobia. In subchronic studies, orally administered osthole in Wistar rats at 5–50 mg/kg bw for 45 days, caused pulmonary haemorrhage and mild reno and hepato-inflammation (Shokoohinia et al., 2017). Esculetin showed a low acute toxicity with an oral LD_50_ of over 2000 mg/kg bw, the intraperitoneal LD_50_ being 1,450 mg/kg bw (Tubaro et al., 1988). Orally administered psoralen in rats (80 mg/kg bw) caused cholestatic liver injuries (Wang et al., 2019).

## 5 Conclusion

Several African medicinal plants are sources of various coumarins which possess several biological activities. Coumarins generally have several therapeutic applications. Most of the coumarins in this review (46.7%) had some antimicrobial activity. Six coumarins: osthole, imperatorin/umbelliferon, pseudocordatolide C & calanolide, chartreusin and esculetin had either antitumor or anticancer activity. They had varying levels and types of toxicity ranging from inhibiting blood clotting as anticoagulants, cytotoxic effects, causing hyperventilation, tremor, and photophobia. Such plants need to be conserved because of their potential as sources of coumarins and other drugs. This calls for more research into their safety and efficacy because of their wide spread applications as therapeutic agents.
